# Integrative single-cell and spatial transcriptomic analysis reveals 4-HNE-related stromal remodeling in lung adenocarcinoma

**DOI:** 10.1016/j.tranon.2026.102995

**Published:** 2026-08-29

**Authors:** Pei Zhang, Ke Ma, Yue Yu, Xu-Chen Cao, Yang Lyu

**Affiliations:** a Department of Intensive Care Unit, Tianjin Medical University Cancer Institute & Hospital, National Clinical Research Center for Cancer, Tianjin’s Clinical Research Center for Cancer, Key Laboratory of Cancer Prevention and Therapy, Huan-Hu-Xi Road, He-Xi District, Tianjin, 300060, China; b The First Department of Breast Cancer, Tianjin Medical University Cancer Institute and Hospital, National Clinical Research Center for Cancer, Key Laboratory of Cancer Prevention and Therapy, Tianjin, Tianjin's Clinical Research Center for Cancer, Huan-Hu-Xi Road, He-Xi District, Tianjin, 300060, China

**Keywords:** Lung adenocarcinoma, 4-HNE, Lipid peroxidation, Single-cell RNA sequencing, Spatial transcriptomics, Tumor microenvironment, RRM2

## Abstract

**Background:**

4-HNE is a lipid peroxidation product involved in oxidative stress, tumor microenvironment remodeling, and tumor progression. This study aimed to characterize the cellular distribution, prognostic relevance, and functional roles of 4-HNE-related molecular features in lung adenocarcinoma.

**Methods:**

4-HNE-related targets were collected from ChEMBL and SwissTargetPrediction, followed by enrichment, differential expression, and survival analyses in TCGA-LUAD. A HNE_score was constructed using single-cell RNA-seq data. Cell-cell communication, fibroblast trajectory, transcription factor activity, spatial transcriptomics, machine learning, molecular docking, virtual knockout, and EdU assays were further performed.

**Results:**

A total of 1,280 4-HNE-related candidate targets were identified and were mainly associated with xenobiotic response, PPAR signaling, estrogen signaling, and chemical carcinogenesis-related pathways. At the single-cell level, higher HNE_score values were mainly observed in macrophage, epithelial, and fibroblast populations. The HNE_high state was associated with enhanced stromal communication and a matrix-remodeling pattern involving fibroblasts, epithelial cells, and myeloid cells, together with enrichment of CTHRC1-positive myofibroblastic cancer-associated fibroblast features. RRM2 and CYP4B1 were identified as stable HNE_score-related prognostic candidates with opposite prognostic associations. RRM2 knockdown reduced DNA synthesis and proliferation in PC9 cells, supporting its functional relevance in lung adenocarcinoma.

**Conclusion:**

This study provides a multilayered map of 4-HNE-related cellular states in lung adenocarcinoma and links 4-HNE activity to stromal remodeling, altered cell-cell communication, and prognostic gene screening. RRM2 and CYP4B1 may represent key HNE-related prognostic candidates, with RRM2 showing functional relevance in tumor cell proliferation.

## Highlights

• 4-HNE activity was mapped in LUAD at single-cell resolution.
• High HNE activity enriched macrophage, epithelial and fibroblast regions.
• HNE-high cells showed fibroblast-centered stromal communication.
• HNE-high fibroblasts were linked to CTHRC1+ myofibroblastic CAFs.
• RRM2 and CYP4B1 were identified as HNE-related prognostic genes.

## Graphical abstract

**Figure:**
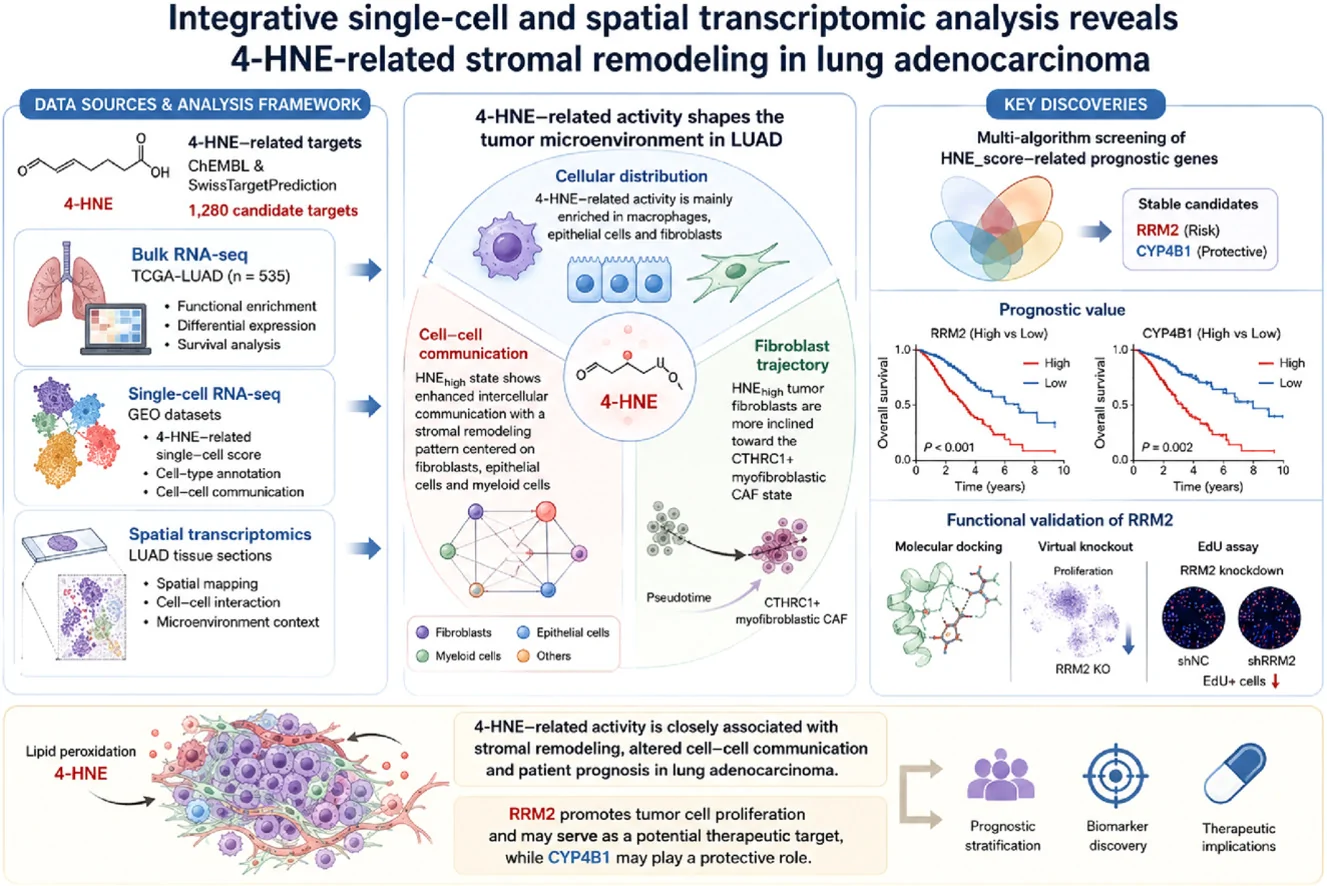

## Introduction

Oxidative stress and lipid peroxidation are common metabolic stress events in tumor tissues[1]. Rapid tumor cell proliferation, hypoxia, inflammatory responses, and mitochondrial dysfunction can promote the accumulation of reactive oxygen species, which further drives membrane lipid peroxidation[1,2]. Lipid peroxidation products are not merely terminal indicators of cellular injury. Some reactive aldehydes can diffuse to neighboring cells and influence cellular behavior through protein adduction, signal transduction, and transcriptional regulation[3]. Therefore, lipid peroxidation-related molecules may provide a link between metabolic stress, tumor microenvironment remodeling, and disease progression[4].

4-HNE is a representative reactive aldehyde generated during lipid peroxidation[5]. Owing to its strong electrophilic property, 4-HNE can react with cysteine, histidine, and lysine residues in proteins, thereby altering protein function and cellular signaling states. In different biological contexts, 4-HNE has been implicated in the regulation of cell proliferation, apoptosis, inflammatory responses, metabolic adaptation, and extracellular matrix remodeling[6,7]. In tumors, the role of 4-HNE may be context dependent. High levels of 4-HNE can induce cytotoxic stress and cellular damage, whereas persistent or locally accumulated 4-HNE-related signals may contribute to adaptive tumor cell responses and microenvironmental remodeling[7,8]. However, in lung adenocarcinoma, the cellular distribution of 4-HNE-related molecular features and their associations with patient prognosis and tumor microenvironment states remain insufficiently characterized.

Lung adenocarcinoma is marked by pronounced cellular heterogeneity[9]. In addition to malignant epithelial cells, macrophages, T cells, fibroblasts, endothelial cells, and other stromal components together form a complex tumor ecosystem[10]. Different cell populations may respond differently to oxidative stress and lipid peroxidation signals, suggesting that 4-HNE-related transcriptional features may show clear cell-type preference[11]. Fibroblasts and cancer-associated fibroblast states are of particular interest because they can reshape tumor tissue architecture through extracellular matrix deposition, ligand-receptor interactions, and transcriptional regulatory programs[12,13]. Although bulk transcriptomic analyses can capture overall expression changes, they cannot determine whether these changes arise from tumor cells, immune cells, or stromal cells[14]. Thus, it is necessary to reassess 4-HNE-related activity in lung adenocarcinoma at single-cell resolution.

Single-cell RNA sequencing and spatial transcriptomics provide a useful framework for addressing this question[15]. Single-cell data enable the evaluation of 4-HNE-related gene set activity across distinct cell types and allow the identification of cellular states associated with high HNE activity. Spatial transcriptomics further supports the localization of key candidate genes and cell-type features within tissue sections[16]. In parallel, network toxicology, machine learning, and survival analysis can help screen prognostically relevant genes from 4-HNE-related targets and connect molecular targets, cellular states, and patient outcomes.

In this study, a multilayered analysis was performed to investigate 4-HNE-related molecular features in lung adenocarcinoma. First, candidate 4-HNE-related targets were collected from the ChEMBL database and SwissTargetPrediction, followed by differential expression and prognostic screening using the TCGA-LUAD cohort. A HNE_score was then constructed using single-cell RNA-seq data to characterize its distribution across major cell types, tissue sources, and HNE activity groups. CellChat, Monocle2, and SCENIC analyses were further used to examine cell-cell communication, fibroblast trajectory states, and transcription factor regulatory patterns. Based on these analyses, multiple machine learning models were applied to identify HNE_score-related prognostic candidate genes, followed by molecular docking, virtual knockout analysis, spatial transcriptomics, and EdU assays for further validation. Through this integrative strategy, this study aimed to clarify the potential role of 4-HNE-related activity in tumor microenvironment remodeling and prognostic feature formation in lung adenocarcinoma.

## Methods

### Collection and standardization of 4-HNE-related targets

4-Hydroxy-2-nonenal (4-HNE) was used as the compound of interest, and its potential targets were collected from the ChEMBL database[17] and the SwissTargetPrediction platform[18]. Targets retrieved from ChEMBL were regarded as experimentally curated bioactivity-associated candidates, whereas those obtained from SwissTargetPrediction were considered computationally predicted targets based on chemical similarity and known ligand–target relationships. Accordingly, the combined gene set was operationally defined as a set of candidate molecular features potentially associated with 4-HNE, rather than a definitive collection of experimentally validated direct 4-HNE-binding targets. For targets obtained from ChEMBL, only records annotated as Homo sapiens were retained when species information was available. UniProt[19] accessions were then extracted and converted into official gene symbols using the R packages AnnotationDbi and org.Hs.eg.db. Duplicated entries and isoform-specific suffixes were removed during this process. Targets predicted by SwissTargetPrediction were also converted into standard gene symbol format. Finally, the union of target genes from the two sources was taken after removing duplicates, generating the final 4-HNE-related candidate target set for subsequent network toxicology analysis.

### Functional enrichment analysis of 4-HNE-related targets

GO-BP and KEGG[20] enrichment analyses were performed separately for the union and intersecting genes of 4-HNE-related targets. Gene symbols were converted into ENTREZID using the clusterProfiler package and annotated based on the org.Hs.eg.db database. P values were adjusted using the Benjamini-Hochberg method, and the top-ranked enriched terms were visualized using Sankey and bubble plots.

### Differential expression analysis of 4-HNE-related genes

Based on the TCGA-LUAD TPM expression matrix, differential expression of the union genes of 4-HNE-related targets was compared between tumor and normal tissues. Expression data were transformed as log2(TPM+1), and differential analysis was performed using the limma package. Genes with an adjusted P value < 0.01 and |logFC| > 1.5 were considered differentially expressed. The expression matrix of these genes was further standardized by Z-score transformation and visualized as a heatmap.

### Identification of prognostic 4-HNE-related genes

To identify 4-HNE-related genes with potential prognostic relevance, the differentially expressed genes were matched with the transcriptomic and overall survival data of patients in the TCGA-LUAD training cohort. Genes detected in both the expression matrix and the candidate gene list were retained for further analysis. For each gene, a Cox proportional hazards model was fitted using gene expression as a continuous variable. Hazard ratios, 95% confidence intervals, and P values were calculated to estimate the association between gene expression and overall survival. Genes with P < 0.05 were considered prognosis-associated candidates. According to the direction of the hazard ratio, these genes were further annotated as risk-associated or protective genes.

### Preprocessing of single-cell RNA-seq data and cell population identification

The dataset comprised 20 lung tissue samples from 12 patients, including 10 tumor tissue samples and 10 normal lung tissue samples. After applying the quality-control criteria used in the present study, 111,967 cells were retained for downstream analysis. Sample-level information, including sample ID, patient ID, tissue source, pairing status, the number of cells retained after quality control, sex, age, anatomical site, primary tumor histology, histological subtype, pathological TNM stage, and the availability of major clinical information, is summarized in Supplementary Table S1. The dataset was analyzed using the Seurat[[21], [22], [23]] package. Expression matrices generated by CellRanger were imported for each sample, and clinical and tissue-source information was matched to cells according to sample IDs. During quality control, cells were retained when they met the following criteria: 500 < nFeature_RNA < 7500, 1000 < nCount_RNA < 80000, mitochondrial gene percentage < 25%, and hemoglobin gene percentage < 5%. After filtering, the expression data were normalized using the LogNormalize method, and the top 3,000 highly variable genes were selected for downstream analysis. The normalized data were then scaled, with sequencing depth, mitochondrial gene percentage, and cell-cycle effects regressed out. Principal component analysis was performed for dimensionality reduction, and Harmony was used to reduce batch effects across samples. The Harmony-corrected space was used to construct the cell-neighbor graph, perform clustering, and generate UMAP embeddings. Clustering was conducted using the first 20 Harmony dimensions. Marker genes for each cluster were identified with the Wilcoxon rank-sum test, and major cell populations were assigned based on canonical marker genes.

### Construction of the HNE_score

Prognosis-related 4-HNE differentially expressed genes identified by Cox analysis were used as the input gene set. Genes detected in the single-cell expression matrix were retained for scoring. Gene-set activity was calculated for each cell using the irGSEA[24] package with multiple algorithms, including AUCell, UCell, singscore, ssGSEA, zscore, and AddModuleScore. Because higher values from all six algorithms represented stronger gene-set activity, no reversal of score direction was required. The score generated by each algorithm was independently rescaled to a 0–1 range using min–max normalization to reduce differences in scale and distribution across methods. The final HNE_score for each cell was calculated as the equally weighted arithmetic mean of the six normalized scores, as follows: HNE_score = (AUCell_norm + UCell_norm + singscore_norm + ssGSEA_norm + zscore_norm + AddModuleScore_norm)/6. Cells were then divided into HNE_high and HNE_low groups according to the median HNE_score for subsequent analyses. The median was selected as an objective cutoff to reduce bias from subjective threshold selection and to maintain relatively balanced cell numbers between the two groups.

### Comparative analysis of cell-cell communication between HNE groups

Cell-cell communication between HNE_low and HNE_high groups was analyzed using the CellChat[22,25] package. The single-cell object was divided according to HNE_group, and major cell types were used as cell identities. CellChatDB.human was used as the reference database to infer ligand-receptor interactions. After identifying overexpressed genes and overexpressed interactions, intercellular communication probabilities were calculated, and interactions involving fewer than 10 cells were filtered out. Signaling communication networks were inferred separately for the two groups, and the CellChat objects were then merged for comparative analysis. The total number and overall strength of interactions were compared between HNE_low and HNE_high groups. Differential interaction networks, signaling sender and receiver patterns, and pathway-level information flow were further evaluated to characterize changes in intercellular communication associated with 4-HNE-related activity.

### Trajectory inference of fibroblast transcriptional states

To investigate transcriptional state changes among fibroblast subsets, annotated fibroblasts were extracted and subjected to pseudotime analysis using Monocle2[26]. A CellDataSet object was generated from the RNA counts matrix, followed by size-factor estimation, dispersion estimation, and gene detection. Genes expressed in at least 10 cells were retained for downstream analysis. Differentially expressed genes across fibroblast subsets were then identified, and the top-ranked genes were selected as ordering genes for trajectory construction. Dimensionality reduction was performed using DDRTree, and cells were ordered along the inferred trajectory with the orderCells function. To aid biological interpretation of the trajectory direction, the state enriched for ADH1B+ resident fibroblasts was set as the root state. The resulting trajectory was visualized according to cell state, fibroblast subset, HNE group, and tissue source. Genes significantly associated with pseudotime were further identified, and a pseudotime heatmap was generated to display dynamic expression patterns along the trajectory.

### SCENIC-based regulon activity analysis

To assess differences in transcriptional regulation across HNE activity states, SCENIC[27] analysis was performed at the single-cell level. Based on HNE_group, 100 cells were randomly sampled without replacement from each of the HNE_low and HNE_high groups, resulting in a balanced input of 200 cells. A random seed of 123 was set before sampling to ensure reproducibility. Repeated subsampling or formal stability assessment was not performed; therefore, the SCENIC analysis was primarily used to explore transcriptional regulatory trends associated with different HNE states rather than to establish definitive regulatory differences. The RNA counts matrix of the sampled cells was used as input. The RNA counts matrix was used as input. After gene filtering, the expression matrix was log2-transformed for downstream analysis. Potential transcription factor-target gene regulatory networks were then inferred from gene co-expression patterns using the GENIE3 algorithm, followed by regulon refinement with hgnc annotations and cisTarget databases. Regulon activity in each cell was quantified using AUCell. Differences in regulon activity were then compared between HNE_low and HNE_high cells, and representative regulons with higher activity in each group were selected. Single-cell heatmaps, average activity heatmaps, differential scatter plots, violin plots, and UMAP feature plots were used to visualize changes in transcription factor regulatory activity between the two HNE groups.

### Screening of HNE_score-correlated genes

To identify candidate genes associated with single-cell HNE activity, HNE_score values and the RNA expression matrix were extracted from the Seurat object for correlation analysis. Cells with missing or invalid HNE_score values were removed. Genes expressed in at least 10 cells and with a mean expression level greater than 0 were retained. Pearson correlation analysis was then performed between each gene and HNE_score, and corresponding P values were calculated. Multiple testing correction was conducted using the Benjamini-Hochberg method. Genes were ranked by correlation coefficient, and the top 300 genes positively correlated with HNE_score were selected as candidate genes for subsequent construction of HNE-related prognostic models. Their correlation strength and statistical significance were visualized using a lollipop plot.

### Machine learning-based construction of HNE-related prognostic models

Based on the top 300 genes positively correlated with the HNE_score, HNE-related prognostic models were further constructed. Candidate genes were first matched with the expression matrix of the TCGA-LUAD training cohort and integrated with overall survival time and survival status. Expression data were log2-transformed when necessary, standardized, and filtered to remove genes with no expression variation before model construction. Multiple machine learning and survival modeling approaches were applied for feature selection and risk prediction, including LASSO-Cox, random survival forest, Boruta-like survival feature selection, GBM-Cox, and ABESS-Cox models. The LASSO-Cox model used cross-validation to determine the optimal penalty parameter. Random survival forest and GBM-Cox models selected candidate genes according to variable importance. The Boruta-like method evaluated feature stability using shadow variables, while the ABESS-Cox model was used for sparse prognostic feature selection. Risk scores were calculated separately for each model, and patients were divided into high- and low-risk groups according to the median risk score. To compare model performance, the C-index of each model was calculated in the training cohort. Gene sets selected by different methods were then summarized, and overlap analysis together with Venn diagrams was used to identify HNE-related prognostic genes that were repeatedly retained across models. This strategy was designed to prioritize feature stability and reproducibility across different modeling frameworks rather than relying solely on the predictive performance of a single model.

### Virtual knockout analysis based on scTenifoldKnk

The scTenifoldKnk[28] package was used to simulate transcriptional regulatory changes after virtual knockout of key genes at the single-cell level. A gene regulatory network was constructed based on the RNA counts matrix, and network perturbations caused by virtual knockout of the target gene were then evaluated. Genes showing marked perturbation after virtual knockout were extracted for GO enrichment analysis to explore the potential biological processes associated with the target gene.

### Spatial mapping of target genes and cell-type features

Spatial transcriptomics data were downloaded from the EBI BioStudies/ArrayExpress database under accession number E-MTAB-13530: https://www.ebi.ac.uk/biostudies/arrayexpress/studies/E-MTAB-13530. The dataset contains NSCLC tissue samples generated using the 10x Genomics Visium platform. Raw expression matrices, spatial coordinates, and tissue image files were obtained and used to construct spatial transcriptomics objects, followed by normalization of the expression matrix. To examine the spatial distribution of key genes and cell-type-related features, the expression levels of CYP4B1 and RRM2 in each spatial spot were extracted and mapped onto the corresponding tissue coordinates. In parallel, cell-type feature scores were calculated for spatial spots based on marker gene sets derived from the single-cell analysis, with a focus on epithelial cell- and fibroblast-associated signatures. The expression of target genes and cell-type feature scores were then projected onto the spatial tissue sections to visualize their spatial distribution patterns.

### PC9 cell culture

The human lung adenocarcinoma PC9 cell line was obtained from the Cell Bank of the Chinese Academy of Sciences, Shanghai, China. The identity of the cell line was confirmed according to the supplier-provided information, and the cells were confirmed to be free of mycoplasma contamination before use. Cells were cultured in RPMI-1640 medium supplemented with 10% fetal bovine serum and 1% penicillin-streptomycin and maintained in a humidified incubator at 37°C with 5% CO₂. When cells reached appropriate confluence, they were passaged using trypsin digestion. Cells in the logarithmic growth phase with good morphology were used for subsequent experiments.

### EdU assay for cell proliferatio

Cell proliferation was assessed using an EdU incorporation assay. Treated PC9 cells were seeded into culture plates and incubated until they reached an appropriate density. EdU working solution was then added, and the cells were incubated further. After incubation, the culture medium was removed, and the cells were fixed with paraformaldehyde and permeabilized with Triton X-100. Fluorescent labeling was performed according to the instructions of the EdU detection kit, followed by nuclear counterstaining with DAPI. Two independent siRNAs targeting RRM2, designated siRRM2_1 and siRRM2_2, were used, with si-NC serving as the negative control. Images were captured under a fluorescence microscope, and the EdU-positive rate was calculated as the percentage of EdU-positive nuclei among the total number of DAPI-stained nuclei. The experiment was independently repeated three times.

### Statistical analysis

All statistical analyses were performed using R software. Continuous variables were presented as mean ± standard deviation or median with interquartile range, depending on data distribution. Comparisons between two groups were conducted using Student’s t-test or the Wilcoxon rank-sum test, while comparisons among multiple groups were performed using one-way analysis of variance or the Kruskal-Wallis test. For differential expression analysis, P values were adjusted using the Benjamini-Hochberg method. Pearson correlation analysis was used to evaluate correlations between variables. Prognosis-related genes were screened using univariate Cox regression analysis, and model performance was assessed by the C-index. Cell proliferation experiments were performed with at least three independent replicates, and group differences were analyzed using t-test or one-way analysis of variance according to the experimental design. All statistical tests were two-sided, and P < 0.05 was considered statistically significant.

## Results

### Identification of 4-HNE-related candidate targets

**Fig. 1:**
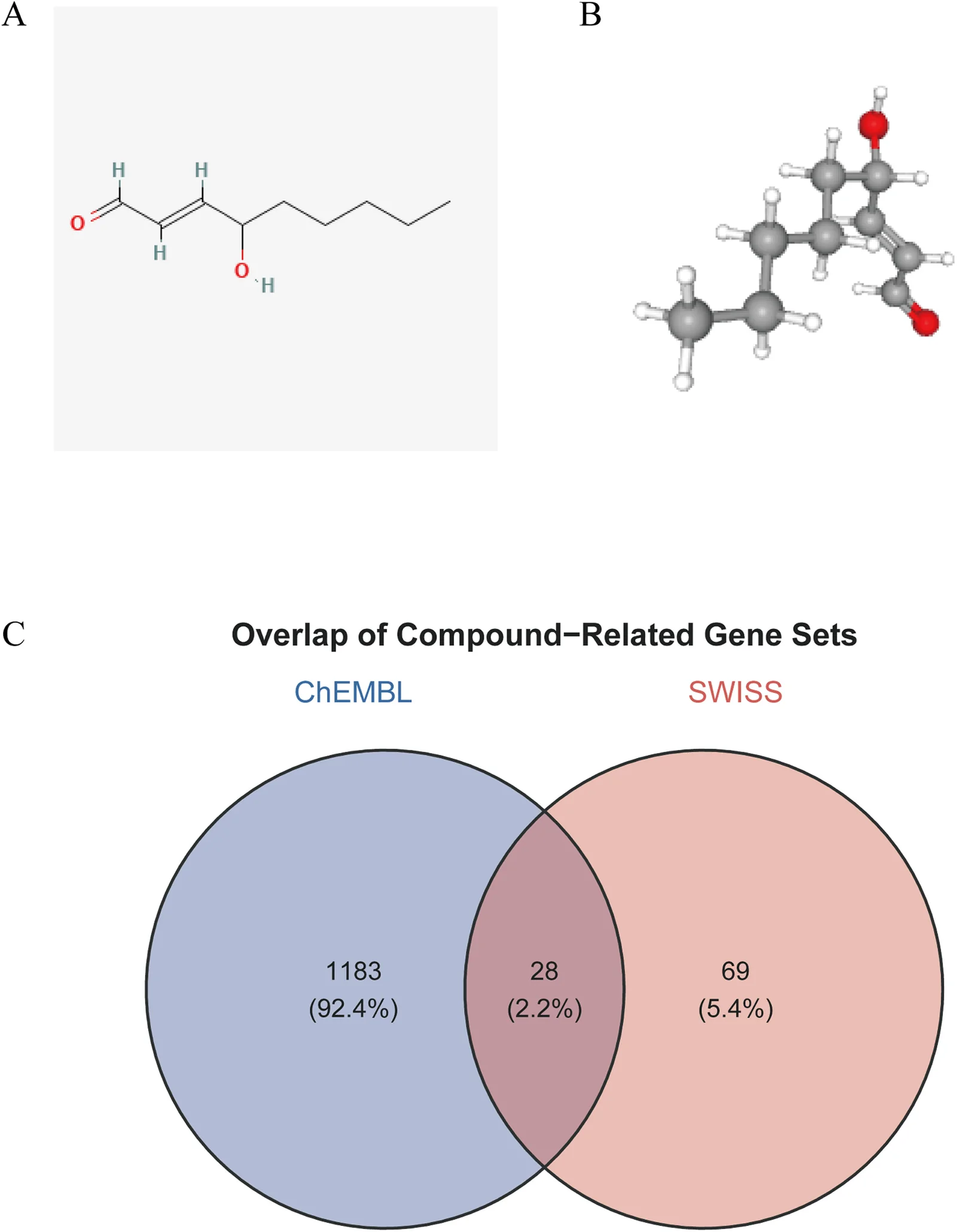
**Molecular structure of 4-HNE and collection of related targets.** (A) Two-dimensional chemical structure of 4-HNE. (B) Three-dimensional molecular conformation of 4-HNE. (C) Venn diagram showing the overlap of 4-HNE-related target genes obtained from the ChEMBL database and the SwissTargetPrediction platform.

To define the potential molecular targets of 4-HNE, its chemical structure and target profiles were first collected and organized. The two-dimensional structure and three-dimensional conformation of 4-HNE were used to show its basic molecular features (Fig. 1A, B). Potential 4-HNE-related targets were then retrieved from the ChEMBL database and the SwissTargetPrediction platform, followed by gene symbol standardization. A total of 1,211 target genes were obtained from ChEMBL, while 97 predicted target genes were identified from SwissTargetPrediction. Among them, 28 genes were shared by both sources, whereas 1,183 genes were specific to ChEMBL and 69 genes were specific to SwissTargetPrediction (Fig. 1C). After taking the union of targets from the two sources, 1,280 4-HNE-related candidate genes were retained for subsequent functional enrichment, differential expression, and prognostic analyses.

### Functional and prognostic characterization of 4-HNE-related genes

**Fig. 2:**
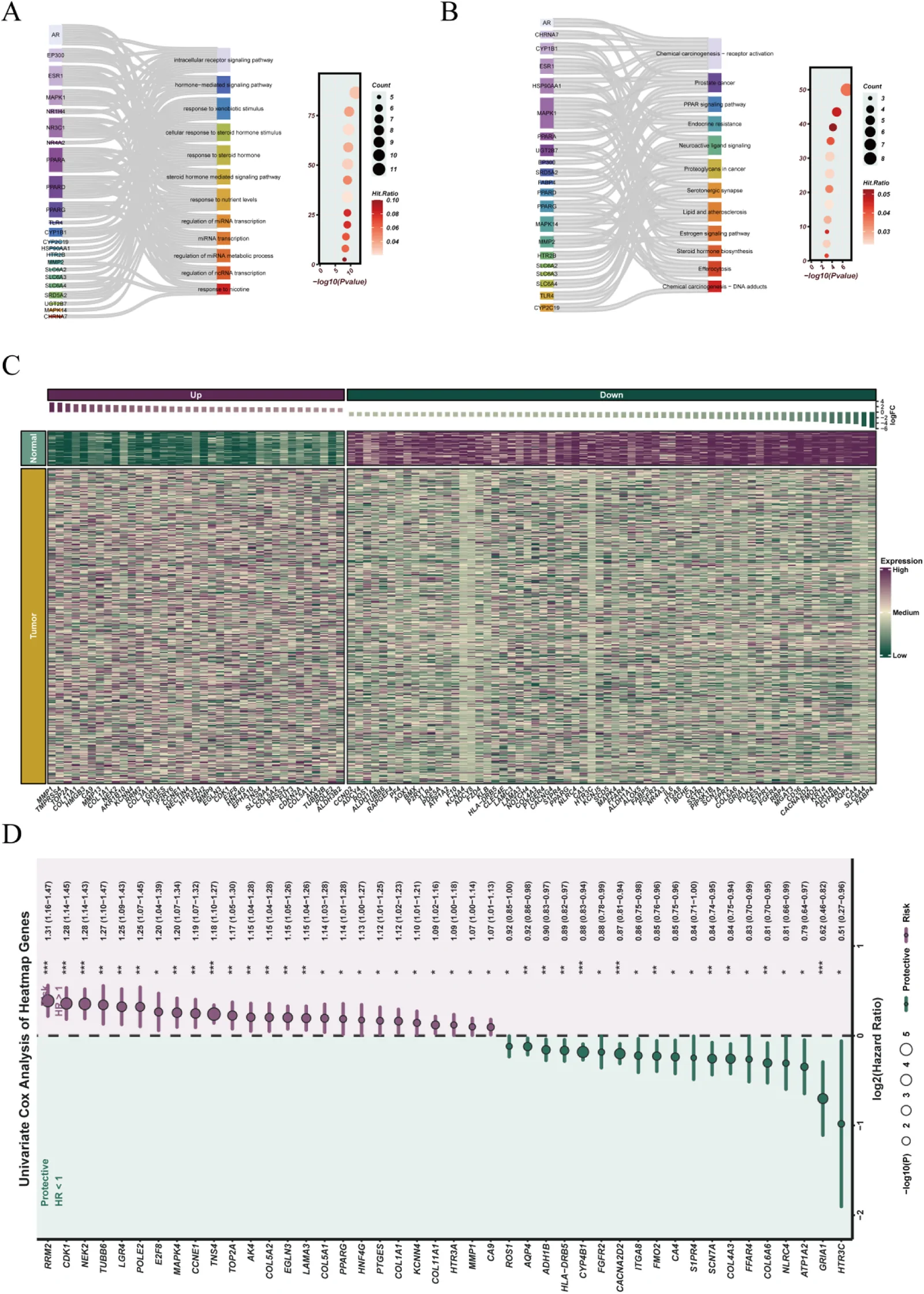
**Functional annotation, differential expression, and prognostic screening of 4-HNE-related genes.** (A) GO-BP enrichment analysis of 4-HNE-related target genes. (B) KEGG pathway enrichment analysis of 4-HNE-related target genes. (C) Heatmap showing the expression pattern of differentially expressed 4-HNE-related genes between normal and tumor tissues in the TCGA-LUAD cohort. (D) Univariate Cox regression analysis of differentially expressed 4-HNE-related genes.

To characterize the biological relevance of 4-HNE-related targets, GO-BP and KEGG enrichment analyses were first performed. GO-BP terms indicated that these genes were mainly associated with cellular responses to xenobiotic stimulus, steroid hormone stimulus, nutrient levels, miRNA-mediated transcriptional regulation, and nuclear receptor-related signaling (Fig. 2A). KEGG analysis further showed that these genes were involved in pathways such as chemical carcinogenesis-receptor activation, PPAR signaling, endocrine resistance, estrogen signaling, lipid and atherosclerosis, and chemical carcinogenesis-DNA adducts (Fig. 2B). The expression profiles of 4-HNE-related genes were then examined in the TCGA-LUAD cohort. Differentially expressed genes showed distinct expression patterns between tumor and normal tissues and were divided into upregulated and downregulated groups according to the direction of logFC (Fig. 2C). Univariate Cox regression analysis further identified a subset of differentially expressed genes associated with overall survival in LUAD. Among them, RRM2, CDK1, NEK2, TUBB6, LGR4, and POLE2 were risk-associated genes, whereas ROS1, AQP4, ADH1B, CYP4B1, FGFR2, and CACNA2D2 were protective genes (Fig. 2D). These findings provided a candidate gene basis for subsequent 4-HNE-related scoring and prognostic modeling.

### Cellular composition and heterogeneity of LUAD tissues

**Fig. 3:**
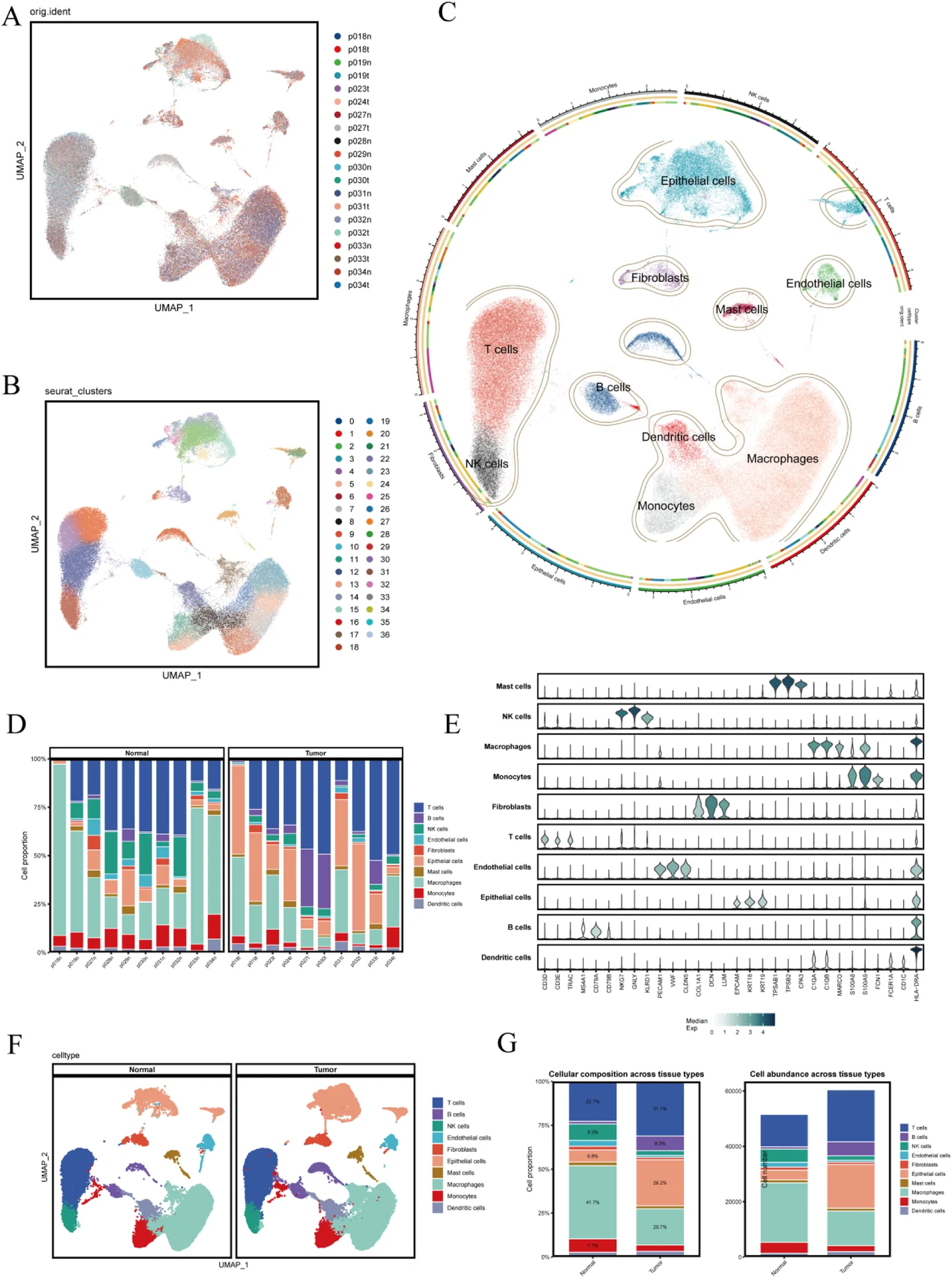
**Single-cell transcriptomic atlas and major cell populations in LUAD.** (A) UMAP distribution of cells colored by sample identity. (B) UMAP visualization of unsupervised Seurat clusters. (C) Circular UMAP visualization of the annotated major cell populations. (D) Sample-level composition of major cell populations in normal and tumor tissues. (E) Expression of canonical marker genes across the annotated major cell populations. (F) UMAP visualization of major cell populations split by tissue source. (G) Cell numbers and relative proportions of major cell populations in normal and tumor tissues.

To characterize the cellular composition of LUAD tissues, all cells were integrated, dimensionally reduced, and clustered. On the UMAP plot, cells from different samples were intermingled rather than forming sample-specific isolated clusters, suggesting that sample-dependent batch effects were largely reduced after integration (Fig. 3A). Unsupervised clustering further separated the cells into multiple transcriptionally distinct clusters, with relatively clear boundaries in the UMAP space, indicating substantial cellular heterogeneity within the dataset (Fig. 3B). Based on cluster-specific marker genes, the main cell populations were then annotated. The LUAD samples contained diverse tumor microenvironment components, including T cells, B cells, NK cells, endothelial cells, fibroblasts, epithelial cells, mast cells, macrophages, monocytes, and dendritic cells (Fig. 3C). These populations together reflected a complex immune, stromal, and epithelial ecosystem in LUAD tissues. Cell composition was next examined at the sample level. The relative abundance of major cell types varied across samples, with some samples showing a higher proportion of immune cells, whereas others were enriched for epithelial or myeloid populations (Fig. 3D). To support the reliability of cell annotation, canonical marker genes were further visualized across the annotated populations. CD3D, CD3E, and TRAC were mainly expressed in T cells; MS4A1, CD79A, and CD79B were enriched in B cells; EPCAM, KRT18, and KRT19 showed higher expression in epithelial cells; COL1A1, DCN, and LUM were primarily detected in fibroblasts; and C1QA, C1QB, and MARCO were mainly expressed in macrophages (Fig. 3E). These marker patterns were consistent with the assigned cell identities. When UMAP plots were split by tissue source, multiple major cell populations were present in both normal and tumor tissues, but their local densities and relative distributions differed between the two tissue types (Fig. 3F). Quantitative composition analysis further showed an increased proportion of epithelial cells in tumor tissues, while macrophages, T cells, fibroblasts, and other microenvironmental populations also showed tissue-dependent differences in abundance (Fig. 3G). Together, these results established a single-cell framework of LUAD cellular composition and provided a basis for subsequent analyses of 4-HNE-related activity across distinct cell populations and tissue contexts.

### Single-cell scoring reveals cell-type-specific 4-HNE-related activity

**Fig. 4:**
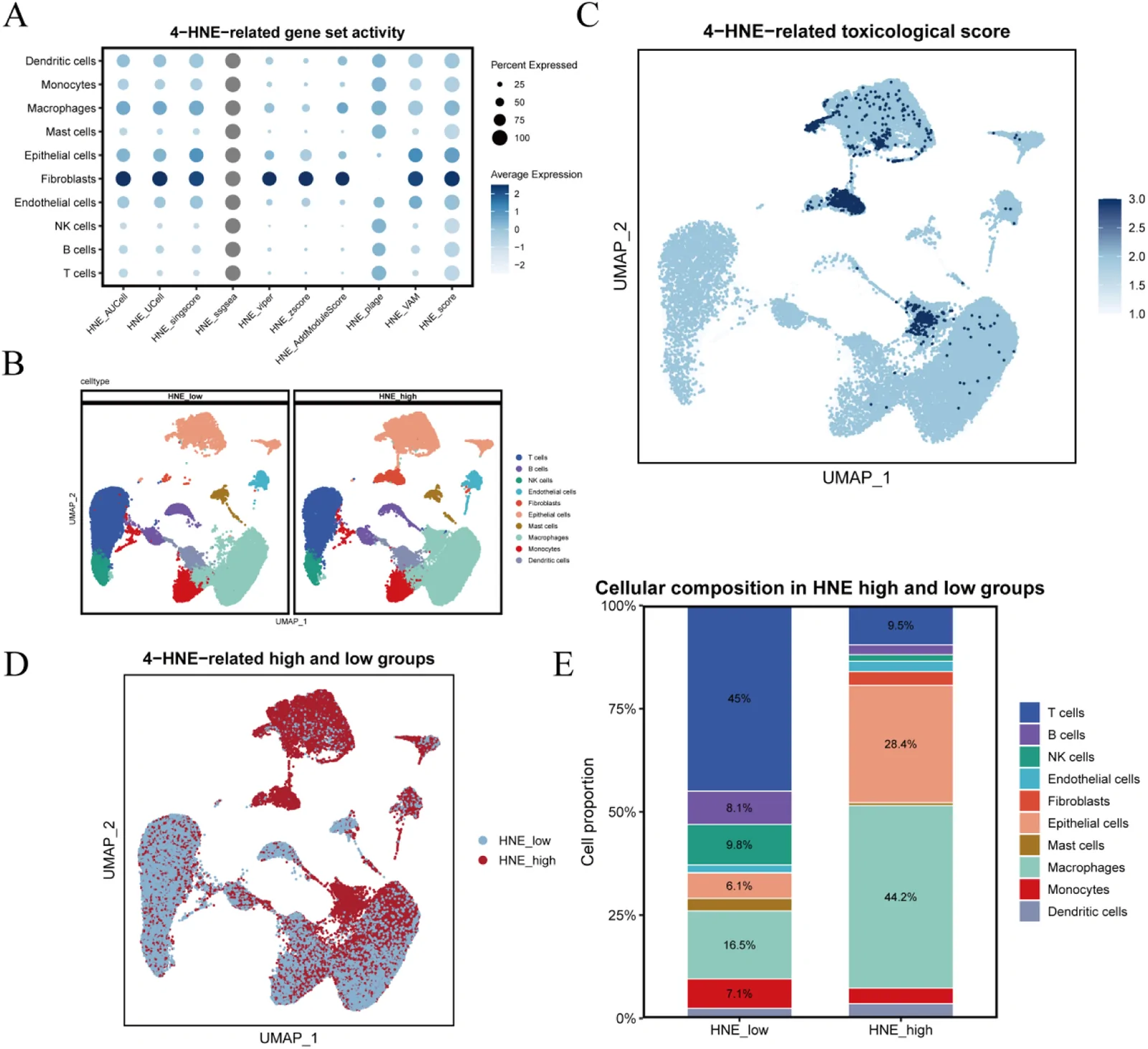
**Construction and cellular distribution of HNE_score.** (A) Distribution of gene-set activity scores generated by six scoring algorithms across the major cell populations. (B) UMAP visualization of major cell populations in the HNE_low and HNE_high groups. (C) UMAP visualization of the continuous HNE_score. (D) UMAP distribution of cells classified into the HNE_low and HNE_high groups. (E) Cell-type composition of the HNE_low and HNE_high groups.

To evaluate the activity of the 4-HNE-related gene set at the single-cell level, multiple gene set scoring methods were applied to calculate 4-HNE-related activity in each cell. The scores generated by different algorithms showed broadly consistent patterns across major cell populations. Fibroblasts, epithelial cells, and some myeloid cells displayed relatively higher 4-HNE-related activity, whereas T cells and B cells showed lower overall scores (Fig. 4A). This suggested that the 4-HNE-related transcriptional program was not evenly distributed across all cell types but was more prominent in specific microenvironmental populations. Cells were then divided into HNE_low and HNE_high groups according to the median HNE_score. Both groups covered multiple major cell populations on the UMAP plots, but HNE_high cells were more concentrated in several defined regions, indicating that elevated 4-HNE-related activity may be linked to specific cell populations or cellular states (Fig. 4B). The continuous distribution of HNE_score further showed that cells with higher scores were mainly located in several local areas of the UMAP space, while low-score cells were more broadly distributed (Fig. 4C). Direct projection of HNE groups onto the UMAP space showed clear enrichment of HNE_high cells in several cell clusters, whereas HNE_low cells were more frequently distributed in immune-cell regions, especially T-cell areas (Fig. 4D). Cell composition analysis further supported this pattern. T cells accounted for a higher proportion in the HNE_low group, while macrophages and epithelial cells were markedly increased in the HNE_high group, accounting for 44.2% and 28.4%, respectively. In contrast, the proportion of T cells decreased to 9.5% in the HNE_high group (Fig. 4E). These findings indicate that 4-HNE-related activity shows clear cell-type preference in the LUAD single-cell atlas and is closely associated with changes in tumor and microenvironmental cell composition.

### 4-HNE-related activity is associated with altered ligand-receptor interactions in the LUAD microenvironment

**Fig. 5:**
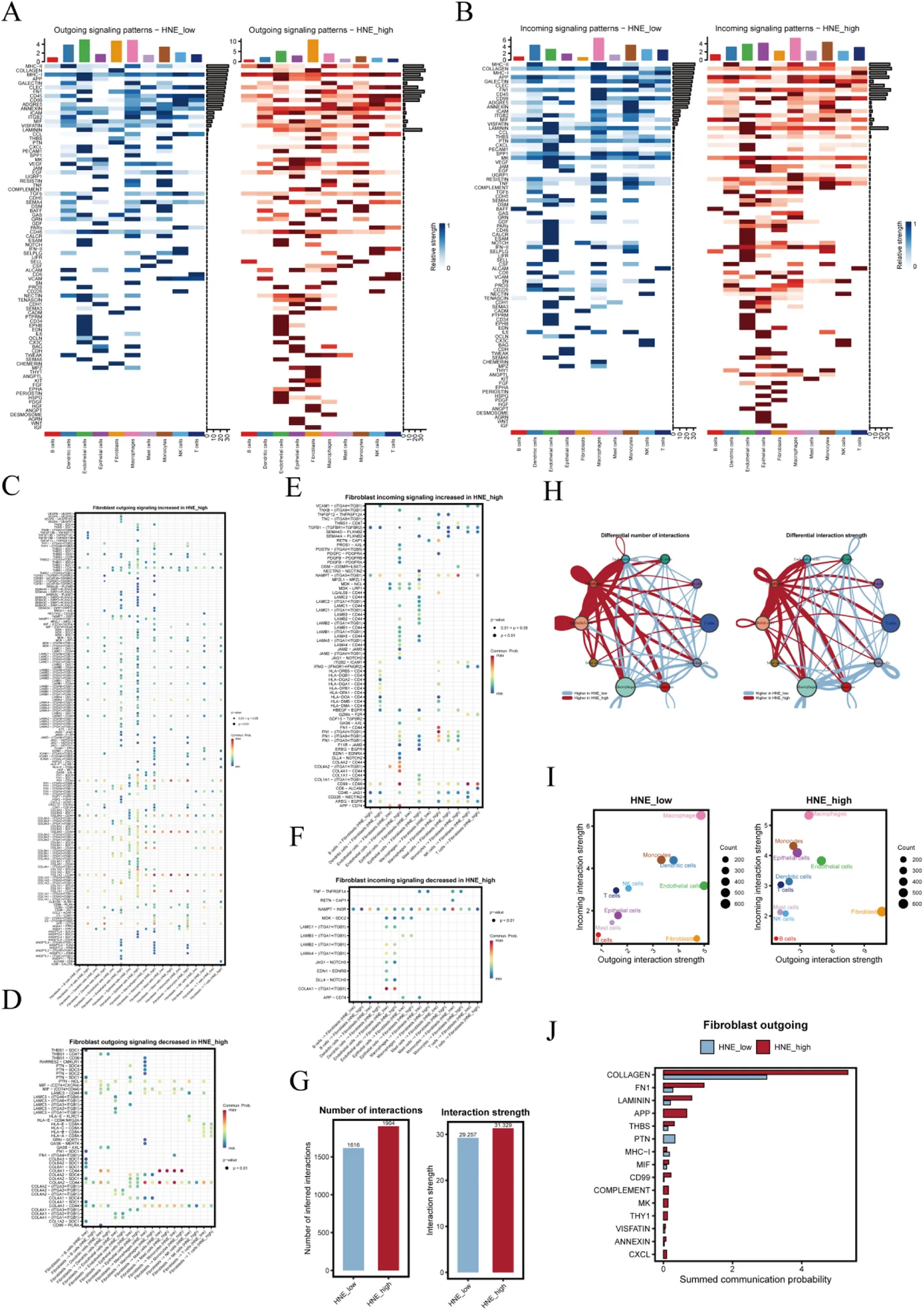
**Comparison of overall and fibroblast-centered intercellular communication between the HNE_low and HNE_high groups.** (A) Heatmaps of outgoing signaling patterns across major cell populations in the HNE_low and HNE_high groups. (B) Heatmaps of incoming signaling patterns across major cell populations in the HNE_low and HNE_high groups. (C) Bubble plot of fibroblast-derived ligand–receptor interactions preferentially detected in the HNE_high group. (D) Bubble plot of fibroblast-derived ligand–receptor interactions preferentially detected in the HNE_low group. (E) Bubble plot of ligand–receptor interactions received by fibroblasts and preferentially detected in the HNE_high group. (F) Bubble plot of ligand–receptor interactions received by fibroblasts and preferentially detected in the HNE_low group. (G) Comparison of the total interaction number and overall interaction strength between the HNE_low and HNE_high groups. (H) Differential networks comparing interaction number and interaction strength between the HNE_high and HNE_low groups. (I) Signaling-role scatter plots showing the outgoing and incoming communication patterns of major cell populations in the HNE_low and HNE_high groups. (J) Comparison of fibroblast outgoing signaling pathway strength between the HNE_low and HNE_high groups.

To systematically compare intercellular communication between the HNE_low and HNE_high states, we examined both the overall communication networks and fibroblast-centered outgoing and incoming interactions using CellChat. The outgoing signaling heatmap showed that fibroblasts, epithelial cells, and macrophages displayed stronger signaling output in the HNE_high group. These signals involved matrix remodeling and cell adhesion-related pathways, including COLLAGEN, FN1, LAMININ, THBS, and APP. In contrast, the HNE_low group showed a more dispersed outgoing signaling pattern, with immune-cell-related pathways contributing to the communication network (Fig. 5A). The incoming signaling heatmap further showed increased signal reception by fibroblasts and epithelial cells in the HNE_high group, suggesting closer communication between stromal cells and tumor epithelial cells under high HNE activity (Fig. 5B). When fibroblasts were analyzed as signal senders, the HNE_high group showed stronger outgoing ligand-receptor interactions toward epithelial cells, macrophages, monocytes, and T cells, suggesting a broader fibroblast-associated signaling pattern involving both tumor and immune cell populations (Fig. 5C). In contrast, fibroblast outgoing interactions preferentially retained in HNE_low cells were fewer, suggesting that the communication profile of fibroblasts differed between the two HNE activity states rather than simply changing in overall magnitude (Fig. 5D). When fibroblasts were considered as signal receivers, stronger incoming signals from epithelial cells, macrophages, monocytes, and T cells were observed in the HNE_high group, consistent with stronger bidirectional communication involving fibroblasts, tumor cells, and immune compartments (Fig. 5E). The HNE_low group showed fewer fibroblast-directed incoming interactions, further supporting a more active communication state of fibroblasts in the HNE_high condition (Fig. 5F). At the global network level, both the number and strength of cell-cell interactions were higher in the HNE_high group than in the HNE_low group (Fig. 5G). Differential network analysis showed that the interactions increased in HNE_high cells were mainly distributed among epithelial cells, fibroblasts, macrophages, and monocytes, whereas interactions relatively enriched in HNE_low cells were more closely associated with T cells and other immune-cell regions (Fig. 5H). Signaling role analysis also showed that fibroblasts and epithelial cells had stronger outgoing and incoming signaling capacities in the HNE_high group, indicating that these two cell types may serve as key communication nodes under high HNE activity (Fig. 5I). Quantitative comparison of fibroblast outgoing pathways further showed that the COLLAGEN pathway was the most prominent in the HNE_high group. FN1, LAMININ, THBS, and APP pathways also showed clear differences between the two groups (Fig. 5J). These results suggest that high HNE activity is associated with a shift in the LUAD microenvironment from a more dispersed immune-related communication pattern toward a stromal remodeling-related communication program centered on fibroblasts, epithelial cells, and myeloid cells.

### HNE_high tumor fibroblasts show enrichment of myofibroblastic CAF features

**Fig. 6:**
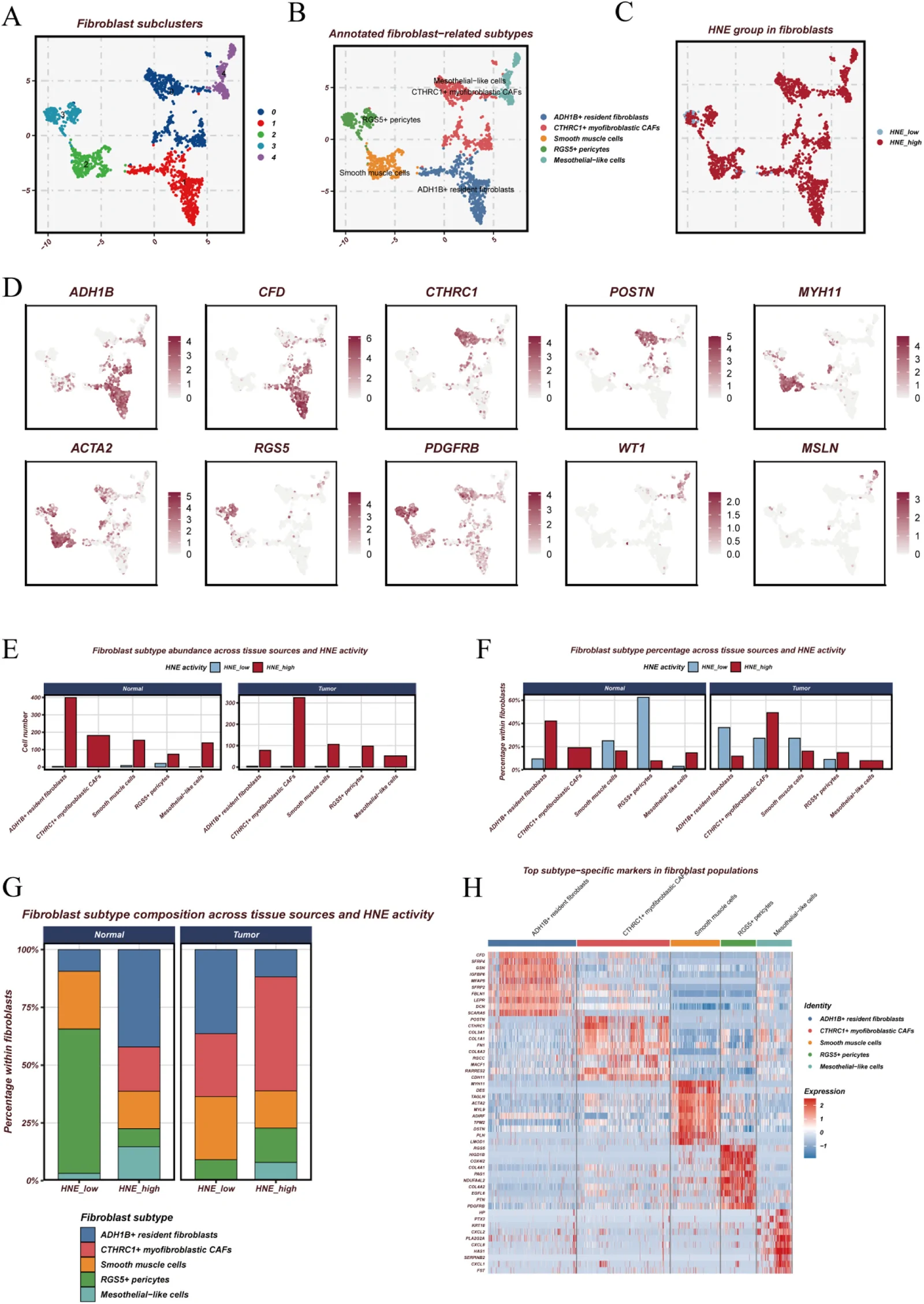
**Fibroblast subpopulations and their distribution across tissue sources and HNE groups.** (A) UMAP visualization of reclustered fibroblasts. (B) UMAP visualization of the annotated fibroblast subpopulations. (C) UMAP distribution of fibroblasts classified into the HNE_low and HNE_high groups. (D) Expression of representative marker genes across fibroblast subpopulations. (E) Cell numbers of fibroblast subpopulations according to tissue source and HNE group. (F) Relative proportions of fibroblast subpopulations according to tissue source and HNE group. (G) Stacked bar plot showing the composition of fibroblast subpopulations across tissue sources and HNE groups. (H) Heatmap of marker gene expression across fibroblast subpopulations.

Fibroblasts were among the major cell populations with relatively high HNE_score and showed prominent involvement in matrix-related communication pathways, including COLLAGEN, FN1, and LAMININ, in the HNE_high state. We therefore further examined this population through subclustering to characterize fibroblast heterogeneity and its distribution across different HNE states. Fibroblasts were extracted from the integrated single-cell dataset and subjected to reclustering, which identified distinct fibroblast-related clusters (Fig. 6A). Based on marker gene expression, these clusters were annotated as ADH1B+ resident fibroblasts, CTHRC1+ myofibroblastic CAFs, smooth muscle cells, RGS5+ pericytes, and mesothelial-like cells (Fig. 6B). Mapping HNE groups onto the fibroblast UMAP revealed that HNE_high fibroblasts were distributed across multiple fibroblast subpopulations, with a clear presence in the CTHRC1+ myofibroblastic CAF region and parts of the ADH1B+ resident fibroblast region. HNE_low fibroblasts were more frequently located in regions corresponding to RGS5+ pericytes and smooth muscle cells (Fig. 6C). Representative marker genes further supported the subtype annotation. ADH1B and CFD marked ADH1B+ resident fibroblasts, CTHRC1 and POSTN corresponded to CTHRC1+ myofibroblastic CAFs, MYH11 and ACTA2 were highly expressed in smooth muscle cells, RGS5 and PDGFRB were enriched in RGS5+ pericytes, and WT1 and MSLN were more consistent with mesothelial-like cells (Fig. 6D). Fibroblast subpopulation abundance was then compared across tissue sources and HNE activity states. In normal tissues, the HNE_high group was mainly composed of ADH1B+ resident fibroblasts, along with increased numbers of CTHRC1+ myofibroblastic CAFs, smooth muscle cells, and mesothelial-like cells. In tumor tissues, CTHRC1+ myofibroblastic CAFs were the most prominent fibroblast subpopulation in the HNE_high group, accompanied by increases in smooth muscle cells and RGS5+ pericytes (Fig. 6E). At the proportional level, RGS5+ pericytes represented a higher fraction in the normal HNE_low group, whereas ADH1B+ resident fibroblasts were increased in the normal HNE_high group. Within tumor tissues, CTHRC1+ myofibroblastic CAFs accounted for the largest fraction in the HNE_high group (Fig. 6F). The stacked composition plot further indicated that tissue source influenced the HNE-related fibroblast landscape. Normal HNE_low tissues were mainly composed of RGS5+ pericytes and smooth muscle cells, whereas ADH1B+ resident fibroblasts represented a larger proportion in normal HNE_high tissues. In tumor tissues, ADH1B+ resident fibroblasts were more abundant in the HNE_low group, while the HNE_high group shifted toward CTHRC1+ myofibroblastic CAFs, with smooth muscle cells and RGS5+ pericytes also retained (Fig. 6G). Finally, the marker gene heatmap showed distinct expression programs across fibroblast subpopulations, supporting the robustness of the subtype classification (Fig. 6H). Overall, fibroblasts in tumor tissues under the HNE_high state showed a stronger myofibroblastic CAF-like composition, suggesting a link between elevated 4-HNE-related activity and stromal remodeling-associated fibroblast states.

### Pseudotime analysis reveals transcriptional continuity among fibroblast states

To further examine the transcriptional relationships among fibroblast subtypes, Monocle2 was used to reconstruct the pseudotime trajectory of fibroblasts. The trajectory showed a continuous distribution with branching structures, and different States occupied distinct regions along the trajectory (Supplementary Figure 1A). When annotated by subtype, ADH1B+ resident fibroblasts, CTHRC1+ myofibroblastic CAFs, smooth muscle cells, RGS5+ pericytes, and mesothelial-like cells displayed distinct but connected positions, suggesting transcriptional continuity among fibroblast states rather than completely separated populations. This inferred trajectory reflects transcriptional continuity among fibroblast states but does not directly establish the direction of cell differentiation or demonstrate a definitive lineage relationship between the identified subtypes. Pseudotime coloring further showed a gradual transition of cells along the trajectory. Mapping HNE groups and tissue sources onto the trajectory indicated that fibroblasts with different HNE activity states and tissue origins occupied different trajectory regions. The heatmap of pseudotime-associated genes revealed stage-dependent expression changes along the trajectory. Some genes showed higher expression at the early pseudotime stage and then gradually declined, whereas others increased at later stages or showed peak expression in intermediate regions, indicating dynamic transcriptional remodeling during fibroblast state transition (Supplementary Figure 1B). Clustering and functional annotation of pseudotime-associated genes showed that different gene modules were related to extracellular matrix organization, response to tumor necrosis factor, muscle contraction, actin-mediated cell contraction, non-canonical Wnt signaling, cytoskeletal regulation, and elastic fiber assembly (Supplementary Figure 1C). These functional programs were closely associated with fibroblast activation, matrix remodeling, and contractile phenotypes. The expression trends of representative genes further supported the trajectory structure. Resident fibroblast-related genes, including ADH1B, CFD, and DCN, showed stage-specific expression along pseudotime. CAF-related genes such as CTHRC1, POSTN, COL1A1, and FAP increased along the trajectory or displayed peak expression in specific trajectory regions. Smooth muscle-like genes, including MYH11, ACTA2, and TAGLN, showed a distinct dynamic pattern (Supplementary Figure 1D). When stratified by HNE group, several genes showed more concentrated expression trends in HNE_high cells, suggesting that 4-HNE-related activity may be linked to specific transcriptional states along the fibroblast trajectory.

### HNE activity is associated with distinct transcription factor regulatory programs

**Fig. 7:**
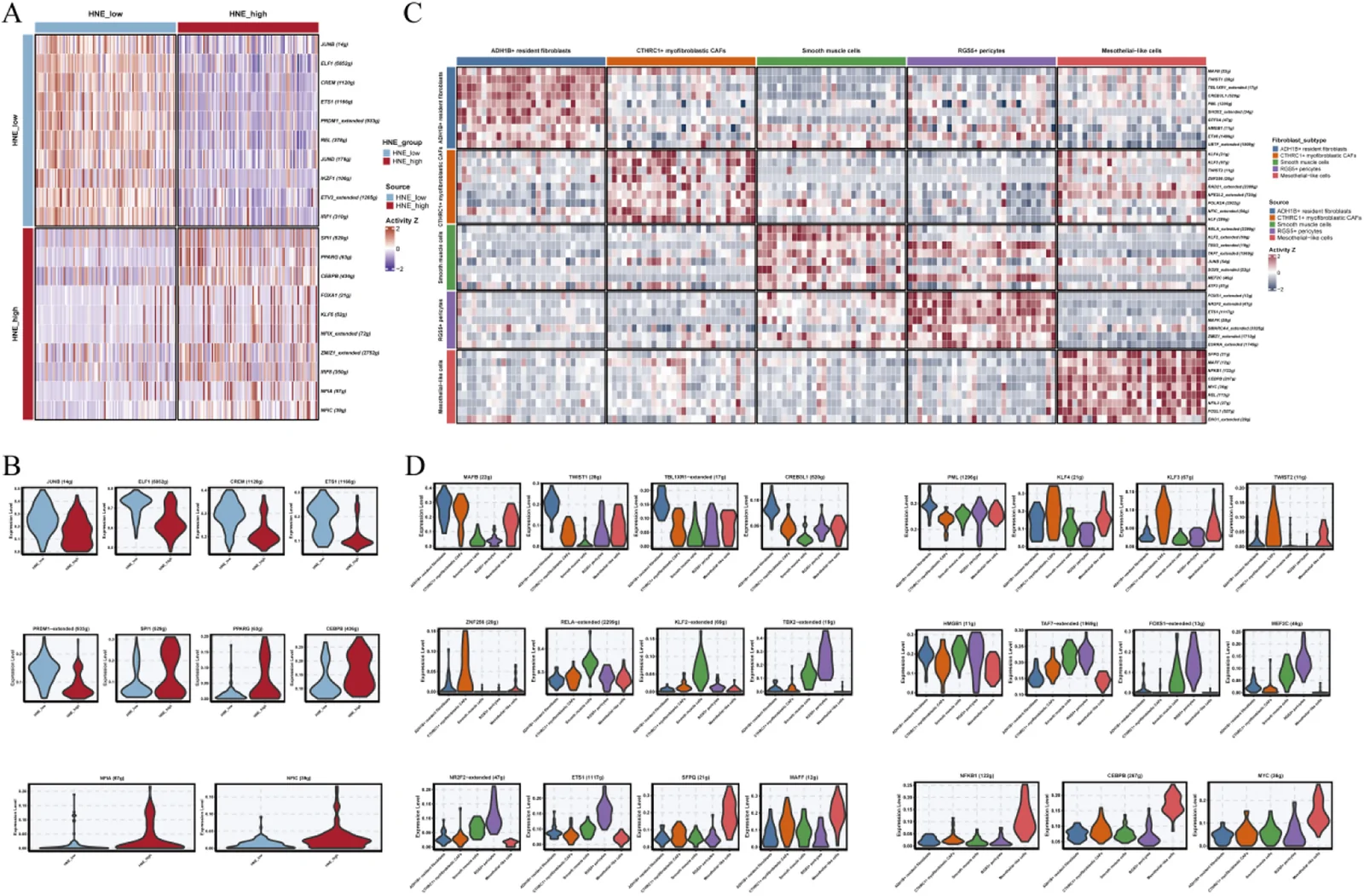
**Analysis of HNE-associated transcription factor regulatory activity.** (A) Single-cell heatmap showing representative regulon activity in HNE_low and HNE_high groups. (B) Violin plots showing representative regulon activity in HNE_low and HNE_high groups. (C) Heatmap showing representative regulon activity across fibroblast subpopulations. (D) Violin plots showing representative regulon activity across fibroblast subpopulations.

To characterize transcriptional regulatory features associated with HNE activity, SCENIC analysis was performed to evaluate regulon activity in HNE_low and HNE_high cells. The single-cell heatmap showed distinct transcription factor regulatory patterns between the two groups. Regulons such as JUNB, ELF1, CREM, ETS1, PRDM1, REL, JUND, IKZF1, ETV3, and IRF1 showed higher activity in the HNE_low group, whereas SPI1, PPARG, CEBPB, FOXA1, KLF5, NFIX, ZMIZ1, IRF8, NFIA, and NFIC were more active in the HNE_high group (Fig. 7A). Violin plots further showed that JUNB, ELF1, CREM, ETS1, and PRDM1-extended were mainly enriched in HNE_low cells, while SPI1, PPARG, CEBPB, NFIA, and NFIC displayed higher activity in HNE_high cells (Fig. 7B). Regulon activity was then examined across fibroblast subpopulations. The heatmap showed that each fibroblast subtype had a distinct regulatory profile. MAFB, TWIST1, and CREB3L1 regulons were more active in ADH1B+ resident fibroblasts; ZNF256, KLF4, KLF3, and TWIST2 showed higher activity in CTHRC1+ myofibroblastic CAFs; NR2F2-extended and ETS1 were prominent in RGS5+ pericytes; and NFKB1, CEBPB, and MYC were more active in mesothelial-like cells (Fig. 7C). Violin plots further confirmed these subtype-associated regulon patterns (Fig. 7D). These results suggest that both HNE activity states and fibroblast subtype differences are accompanied by changes in specific transcription factor regulatory programs.

### Multi-algorithm screening of HNE_score-related prognostic genes

**Fig. 8:**
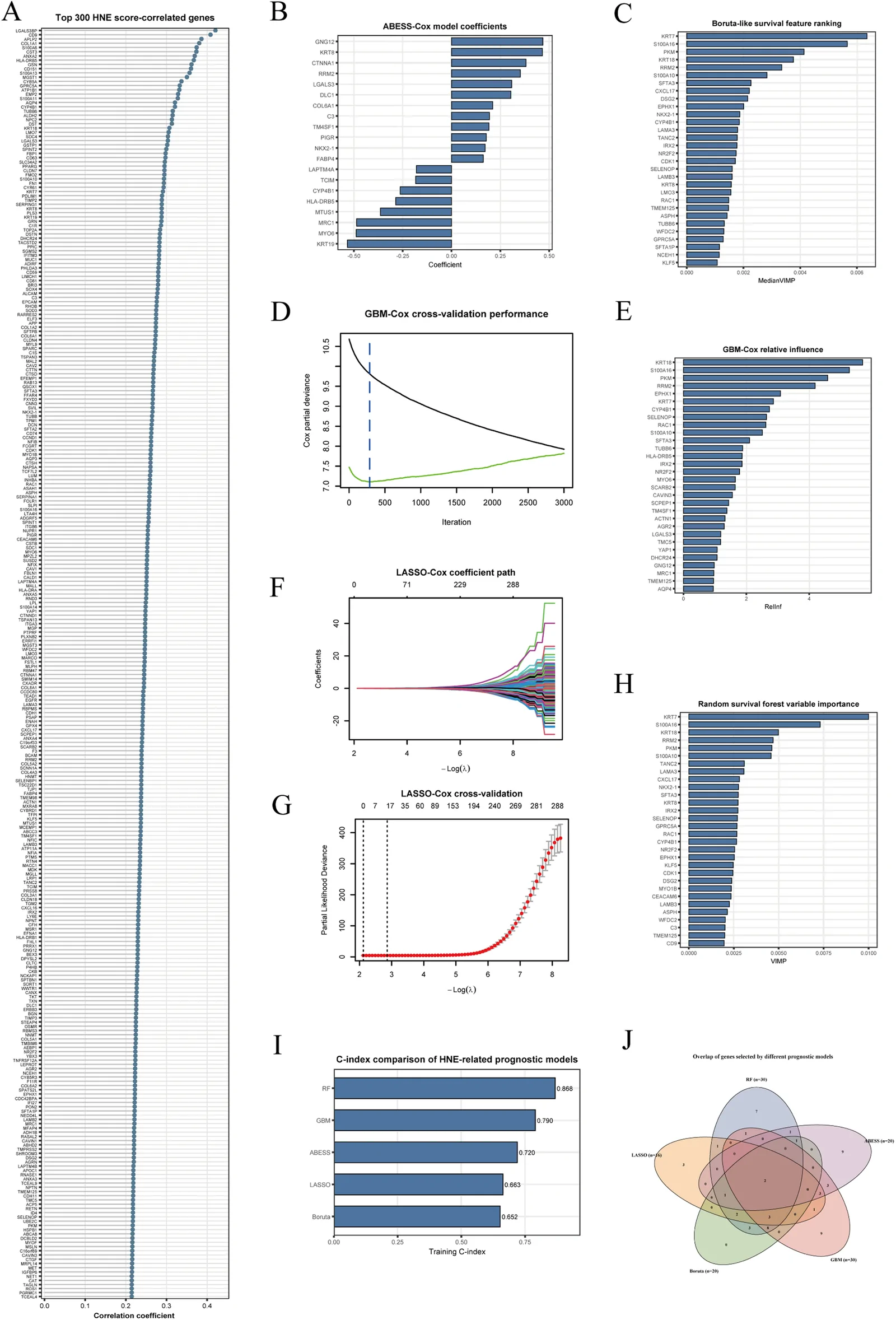
**Construction of prognostic models and feature selection based on HNE_score-correlated genes.** (A) Top 300 genes positively correlated with HNE_score and their correlation coefficients. (B) Regression coefficients of genes selected by the ABESS-Cox model. (C) Top-ranked candidate genes identified by Boruta-like survival feature selection. (D) Cross-validation curve of the GBM-Cox model. (E) Relative influence ranking of candidate genes in the GBM-Cox model. (F) Coefficient path of the LASSO-Cox model. (G) Cross-validation curve of the LASSO-Cox model. (H) Variable importance ranking of candidate genes in the random survival forest model. (I) C-index comparison of different prognostic models in the training cohort. (J) Overlap analysis of gene sets selected by different models.

To clarify the screening workflow, candidate genes were prioritized through a stepwise strategy. First, genome-wide correlations between gene expression and HNE_score were calculated at the single-cell level, and the top 300 positively correlated genes were retained as the initial candidate set (Fig. 8A). These genes were subsequently subjected to feature selection using five machine learning and survival modeling approaches, allowing stable candidates to be identified according to their repeated retention across different algorithms. Multiple algorithms were then applied for prognostic feature selection, including ABESS-Cox, Boruta-like survival feature selection, GBM-Cox, LASSO-Cox, and random survival forest. The ABESS-Cox model retained genes with either positive or negative coefficients. KRT8, CTNNA1, RRM2, LGALS3, and COL6A1 showed positive coefficients, whereas KRT19, MYO6, MRC1, and MTUS1 showed negative coefficients (Fig. 8B). The Boruta-like method ranked genes according to feature stability and variable importance, with KRT7, S100A16, PKM, KRT18, and RRM2 among the top-ranked genes (Fig. 8C). In the GBM-Cox model, cross-validation was used to determine model performance across boosting iterations, and relative influence analysis highlighted KRT8, S100A16, PKM, RRM2, and EPHX1 as highly contributing variables (Fig. 8D, E). In the LASSO-Cox model, gene coefficients gradually shrank as the penalty parameter changed, and cross-validation was used to determine the model parameter (Fig. 8F, G). The random survival forest model ranked genes by variable importance, where KRT7, S100A16, KRT18, RRM2, and PKM again showed high importance (Fig. 8H). Model comparison based on the training cohort showed that the random survival forest model achieved the highest C-index (0.868), followed by GBM-Cox (0.790) and ABESS-Cox (0.720). The C-index values of the LASSO-Cox and Boruta-like models were 0.663 and 0.652, respectively (Fig. 8I). Although the random survival forest model showed the highest C-index in the training cohort, predictive performance was not used as the sole criterion for candidate gene selection. Features identified by a single model may be influenced by its specific learning structure, variable-importance calculation, and sensitivity to the training data. We therefore prioritized genes that were repeatedly retained across different machine learning and survival modeling approaches, with the aim of reducing algorithm-specific selection bias and identifying candidates with greater stability and reproducibility. Overlap analysis of the gene sets selected by different algorithms showed both model-specific genes and shared candidate genes (Fig. 8J). RRM2 and CYP4B1 were repeatedly retained across model intersections and also showed clear prognostic associations. They were therefore prioritized as stable HNE_score-related prognostic candidates for subsequent analyses of expression distribution, spatial localization, and functional relevance.

### RRM2 and CYP4B1 show distinct expression and prognostic patterns

**Fig. 9:**
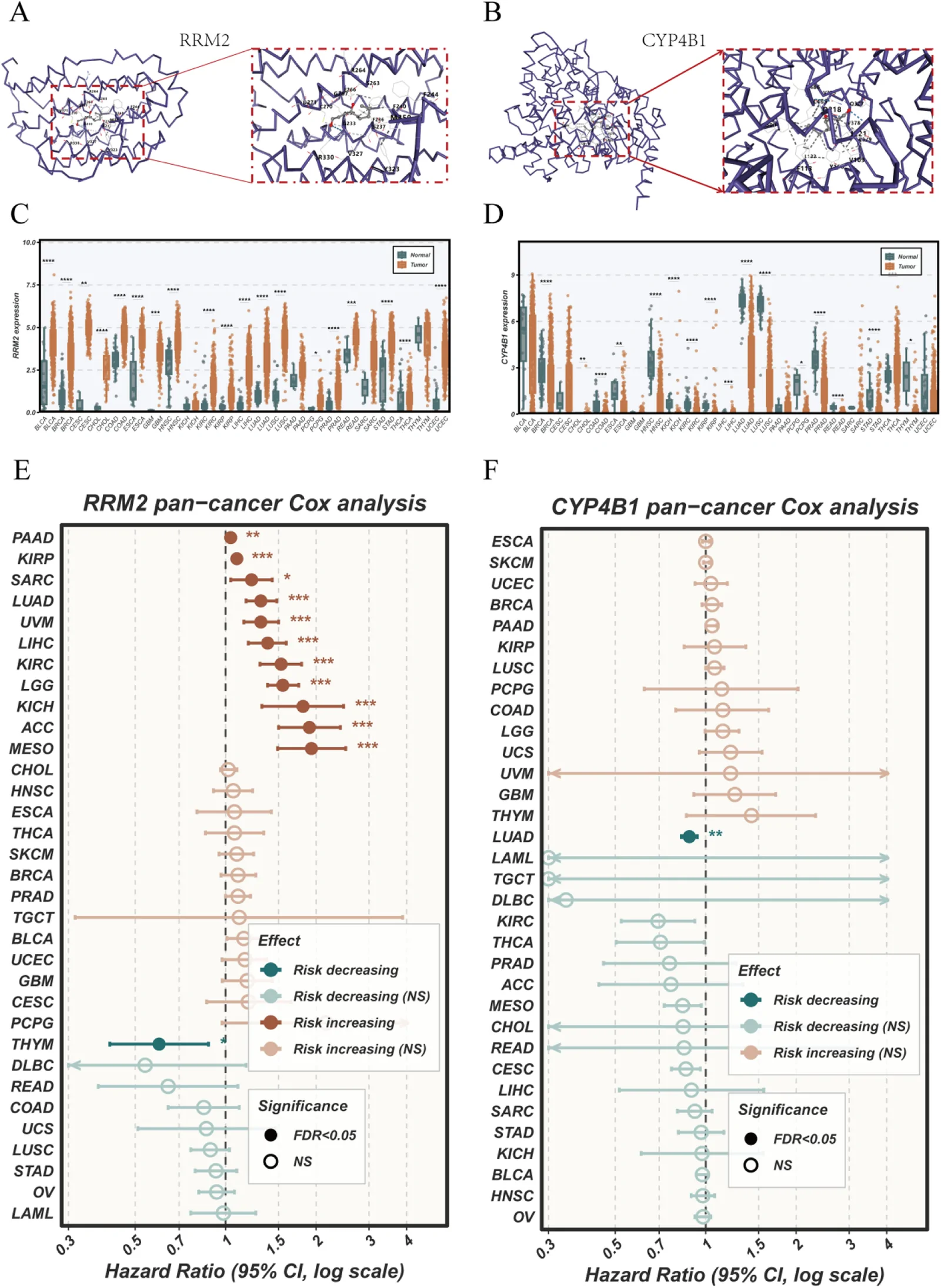
**Molecular docking, pan-cancer expression, and prognostic profiles of RRM2 and CYP4B1.** (A) Molecular docking conformation of 4-HNE with RRM2 and magnified view of the local binding site. (B) Molecular docking conformation of 4-HNE with CYP4B1 and magnified view of the local binding site. (C) Differential expression of RRM2 between tumor and normal tissues across cancer types. (D) Differential expression of CYP4B1 between tumor and normal tissues across cancer types. (E) Univariate Cox regression analysis of RRM2 across cancer types. (F) Univariate Cox regression analysis of CYP4B1 across cancer types.

RRM2 and CYP4B1 were repeatedly retained during multi-algorithm screening; therefore, they were further evaluated from the perspectives of molecular interaction, pan-cancer expression, and prognostic relevance. Molecular docking showed that 4-HNE could fit into a local binding region of the RRM2 protein, with a clear spatial contact pattern in the magnified view (Fig. 9A). Similarly, 4-HNE formed a stable docking pose with CYP4B1, suggesting that the proteins encoded by these two candidate genes may be connected to 4-HNE-related molecular effects (Fig. 9B). The expression patterns of RRM2 and CYP4B1 were then compared across cancer types. RRM2 showed a broad upregulation trend in tumor tissues, with significant differences between tumor and normal tissues in LUAD and several other solid tumors (Fig. 9C). In contrast, CYP4B1 displayed a more cancer-type-dependent expression pattern. Its expression was lower in tumor tissues than in normal tissues in several cancer types, while other cancer types showed changes in different directions. In LUAD, CYP4B1 also showed a significant expression difference between tumor and normal tissues (Fig. 9D). Pan-cancer univariate Cox regression analysis further showed that RRM2 acted mainly as a risk-associated gene across multiple cancer types, with hazard ratios greater than 1 in PAAD, KIRP, SARC, LUAD, UVM, LIHC, KIRC, LGG, KICH, ACC, and MESO (Fig. 9E). In LUAD, RRM2 also showed a hazard ratio greater than 1, indicating that higher RRM2 expression was associated with poorer prognosis. By contrast, the prognostic effect of CYP4B1 was more cancer-type specific and was not significant in most cancer types. In LUAD, CYP4B1 showed a protective association, with a hazard ratio less than 1 and statistical significance (Fig. 9F). These findings indicate that although RRM2 and CYP4B1 were both identified from HNE-related prognostic feature screening, they showed distinct expression patterns and opposite prognostic directions in LUAD.

### Spatial transcriptomics and functional assays validate HNE-related candidate genes

**Fig. 10:**
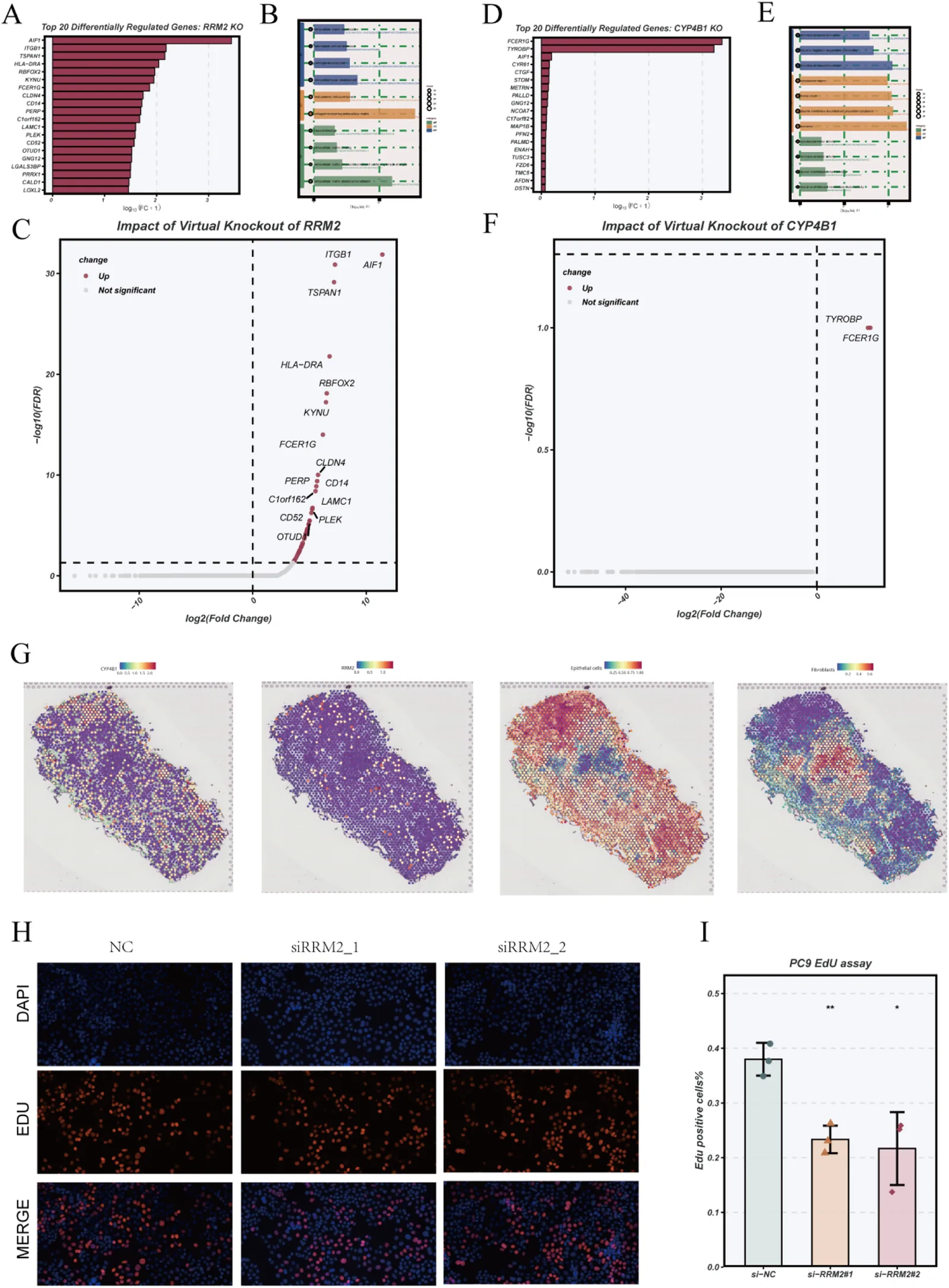
**Virtual knockout, spatial localization, and functional validation of RRM2 and CYP4B1.** (A) Top 20 genes with the strongest perturbation after virtual knockout of RRM2. (B) GO enrichment analysis of perturbed genes associated with RRM2 virtual knockout. (C) Volcano plot showing gene perturbation changes after RRM2 virtual knockout. (D) Top 20 genes with the strongest perturbation after virtual knockout of CYP4B1. (E) GO enrichment analysis of perturbed genes associated with CYP4B1 virtual knockout. (F) Volcano plot showing gene perturbation changes after CYP4B1 virtual knockout. (G) Spatial distribution of CYP4B1 expression, RRM2 expression, epithelial cell-related features, and fibroblast-related features in spatial transcriptomics sections. (H) Representative fluorescence images of EdU incorporation in PC9 cells transfected with si-NC or two independent RRM2-targeting siRNAs, siRRM2_1 and siRRM2_2. (I) Quantification of the EdU-positive rate, calculated as the percentage of EdU-positive nuclei among total DAPI-stained nuclei. Data are presented as the mean ± SD from three independent biological replicates. Statistical significance was assessed using one-way analysis of variance followed by Dunnett’s multiple-comparison test, with each siRRM2 group compared with the si-NC group.

To further evaluate the potential roles of RRM2 and CYP4B1 in single-cell transcriptional networks, virtual knockout analysis was performed using scTenifoldKnk. After virtual knockout of RRM2, genes such as ITGB1, AIF1, TSPAN1, HLA-DRA, RBFOX2, KYNU, FCER1G, CLDN4, and PLEK showed marked perturbation, suggesting that RRM2 depletion may affect genes related to cell adhesion, immune-associated molecules, and epithelial cell states (Fig. 10A). GO enrichment analysis showed that RRM2 knockout-perturbed genes were mainly enriched in extracellular matrix organization, heterotypic cell-cell adhesion, connective tissue development, collagen-containing extracellular matrix, heparin binding, and extracellular matrix structural constituent terms, indicating that RRM2-related network perturbation was closely associated with extracellular matrix and cell adhesion processes (Fig. 10B). The volcano plot further showed that several genes were significantly perturbed after RRM2 virtual knockout, with ITGB1, AIF1, TSPAN1, HLA-DRA, and FCER1G located in the prominent upregulated perturbation region (Fig. 10C).

CYP4B1 was then subjected to virtual knockout analysis. After CYP4B1 virtual knockout, FCER1G, TYROBP, CTSO, STOM, METRNL, NCAT1, CTHRC1, and PIEZO1 ranked among the most strongly perturbed genes (Fig. 10D). GO enrichment analysis showed that CYP4B1-associated perturbed genes were mainly enriched in microtubule bundle formation, cilium- or flagellum-dependent cell motility, microtubule-based movement, motile cilium, axoneme, cytoskeletal motor activity, and microtubule motor activity, suggesting that CYP4B1-related perturbation was more closely related to microtubule, ciliary motility, and cytoskeletal processes (Fig. 10E). The volcano plot showed fewer significantly perturbed genes after CYP4B1 virtual knockout, with TYROBP and FCER1G showing relatively clear upregulated perturbation (Fig. 10F).

Spatial transcriptomics data were further used to examine the spatial distribution of RRM2 and CYP4B1 in tissue sections. CYP4B1 and RRM2 showed heterogeneous expression across spatial spots, with RRM2 displaying relatively weak and focal signals. The epithelial cell-related score was broadly distributed across the section, whereas the fibroblast-related score showed more regional enrichment, indicating that RRM2 and CYP4B1 expression occurred within a spatially heterogeneous cellular microenvironment (Fig. 10G). Finally, EdU assays were performed in PC9 cells using two independent RRM2-targeting siRNAs, siRRM2_1 and siRRM2_2. Compared with the si-NC group, both siRRM2 treatments reduced the number of EdU-positive cells, indicating decreased DNA synthesis activity following RRM2 knockdown (Fig. 10H). The EdU-positive rate was quantified as the percentage of EdU-positive nuclei among total DAPI-stained nuclei. Quantitative data from three independent biological replicates were analyzed using one-way analysis of variance followed by Dunnett’s multiple-comparison test, with each siRRM2 group compared with the si-NC group (Fig. 10I). These findings support a proliferation-promoting role of RRM2 in LUAD cells and suggest that RRM2 may represent a functional candidate gene within the HNE-related prognostic signature.

## Discussion

This study started from 4-HNE, a lipid peroxidation-derived reactive aldehyde, and aimed to address a more specific question: in lung adenocarcinoma, do 4-HNE-related molecular features simply reflect oxidative injury, or do they represent a cellular state associated with tumor microenvironment remodeling and prognostic heterogeneity? By integrating network toxicology, single-cell transcriptomics, spatial transcriptomics, and machine learning, we found that 4-HNE-related activity was not randomly distributed across all cell types. Instead, it was more prominent in epithelial cells, myeloid cells, and fibroblast-related regions. This pattern suggests that 4-HNE-related signaling may reflect a tissue stress and microenvironment remodeling state, rather than a passive consequence of increased lipid peroxidation alone(4, 5).

4-HNE is highly reactive and can influence cellular function through protein adduction and signaling regulation. Previous studies have often discussed 4-HNE in the context of oxidative damage, cytotoxicity, or metabolic disturbance[7]. However, its role in tumor tissues may be more complex. Excessive 4-HNE can induce cellular damage, whereas chronic, local, or sublethal 4-HNE-related responses may promote adaptive changes in tumor cells and surrounding stromal cells. In this study, 4-HNE-related targets were enriched in responses to xenobiotic stimulus, nuclear receptor signaling, PPAR signaling, estrogen signaling, and chemical carcinogenesis-related pathways. These findings indicate that 4-HNE may connect environmental stress responses, lipid metabolism, and transcriptional regulation[29,30]. Therefore, in the context of lung adenocarcinoma, 4-HNE should not be viewed only as a marker of oxidative injury, but also as a potential contributor to cellular state transitions and microenvironmental adaptation.

The single-cell results further emphasized the cell-state dependence of 4-HNE-related activity. HNE_high cells were mainly located in macrophage-, epithelial cell-, and fibroblast-related regions, whereas lymphocyte-related regions showed relatively lower activity. This distribution is biologically plausible. Tumor epithelial cells are major carriers of oxidative stress and metabolic reprogramming[31]. Myeloid cells are highly sensitive to inflammatory and lipid metabolic changes[32]. Fibroblasts directly participate in matrix deposition and tissue remodeling[33]. Thus, the HNE_high state may represent a stress-associated microenvironment jointly shaped by tumor cells, myeloid cells, and stromal cells, rather than a change restricted to a single cell type.

The fibroblast-related findings are one of the key aspects of this study. Under the HNE_high state, cell-cell communication shifted toward matrix-related pathways such as COLLAGEN, FN1, LAMININ, and THBS, and tumor fibroblasts showed a stronger tendency toward the CTHRC1+ myofibroblastic CAF state. CTHRC1+ CAFs are often linked to collagen remodeling, tissue stiffness, cell migration, and an invasive tumor microenvironment[34,35]. These observations suggest that increased 4-HNE-related activity may be involved in the formation of a stromal remodeling niche. In this niche, fibroblasts may not only serve as a source of extracellular matrix, but also regulate epithelial and myeloid cell states through ligand-receptor interactions[36,37]. In other words, 4-HNE-related activity may contribute to lung adenocarcinoma progression through a continuous process involving oxidative stress, matrix remodeling, and enhanced intercellular communication.

Pseudotime analysis also supported the idea that fibroblasts are not a static population. Different fibroblast subtypes were distributed along a connected trajectory, suggesting transcriptional continuity among resident fibroblasts, myofibroblastic CAFs, pericytes, and smooth muscle-like cells. It should be noted that pseudotime analysis does not prove a real differentiation direction. Even so, it provides a useful clue: the HNE_high state was accompanied by more active matrix production, contractile programs, and cytoskeletal remodeling. This was consistent with the enhanced matrix-related signaling observed in the CellChat analysis. Together, these results suggest that 4-HNE-related features may mark a shift of fibroblasts from a relatively homeostatic state toward a remodeling CAF-like state.

Transcription factor regulatory analysis provided another layer of explanation. SPI1, CEBPB, PPARG, FOXA1, and KLF5 showed higher regulon activity in HNE_high cells. These factors are respectively related to myeloid cell states, inflammatory responses, lipid metabolic adaptation, and epithelial transcriptional programs[38,39]. This suggests that HNE_high is not defined by the upregulation of a few genes alone, but may represent a broader regulatory network change shared across multiple cell populations. In particular, the activity of PPARG and CEBPB may connect lipid metabolism, inflammation, and myeloid cell function, and may contribute to the formation of the HNE_high microenvironmental state.

The prognostic modeling results also deserve further interpretation. RRM2 and CYP4B1 were repeatedly retained across multiple algorithms, but their biological implications appeared different. RRM2 is closely related to DNA synthesis and cell proliferation, and its risk-associated role in lung adenocarcinoma is consistent with its known proliferative function. More importantly, RRM2 was not selected from a conventional differential expression analysis alone; it was repeatedly retained from HNE_score-correlated genes across several machine learning models. This suggests that RRM2 may connect high-HNE activity-related cellular states with patient prognosis. The EdU assay further showed that RRM2 knockdown reduced PC9 cell proliferation, supporting its functional relevance. In contrast, CYP4B1 showed a protective association in LUAD, which may be related to lung tissue-specific metabolic functions or the maintenance of epithelial differentiation. From this perspective, RRM2 and CYP4B1 may hypothetically represent two opposing components within HNE-related molecular features: a proliferation-related risk axis and a lung tissue metabolism/differentiation-related protective axis, respectively. This interpretation remains provisional and requires further experimental validation, particularly to determine whether these two genes directly participate in distinct biological responses associated with 4-HNE.

The integrative design of this study has several advantages. Network toxicology provided a starting point for defining 4-HNE-related candidate targets. Single-cell analysis clarified the cellular sources of 4-HNE-related activity. Spatial transcriptomics added tissue localization information. Machine learning and experimental validation further supported the selection of key candidate genes. This strategy helps avoid the limitation of bulk transcriptomic studies, in which gene-level signals are often disconnected from their cellular origins, and places lipid peroxidation-related molecular events back into the context of the lung adenocarcinoma microenvironment.

Several limitations should also be acknowledged. First, HNE_score was constructed from 4-HNE-related gene sets at the transcriptional level, and therefore cannot be considered a direct measurement of intracellular 4-HNE concentration or 4-HNE-protein adducts. Future studies should incorporate 4-HNE immunostaining, lipid peroxidation assays, or spatial metabolomics for further validation. Second, the single-cell and spatial transcriptomic analyses were based on public datasets, with limited sample size and clinical information. This may affect the generalizability of the conclusions. Third, the current experimental validation mainly showed that RRM2 affects PC9 cell proliferation, but it did not establish a direct causal relationship between RRM2 and 4-HNE stimulation. Further experiments involving 4-HNE treatment, RRM2 knockdown or overexpression, matrix-related factor detection, and co-culture systems are needed to clarify whether 4-HNE-related activity influences lung adenocarcinoma progression through RRM2 or CAF-related communication axes. The protective role of CYP4B1 also requires further functional validation. Fourth, the molecular docking analysis only suggests potential structural compatibility between 4-HNE and RRM2 or CYP4B1. It does not demonstrate direct binding, covalent adduct formation, or a direct regulatory relationship in a cellular context. Further biochemical and cell-based experiments are required to determine whether these predicted interactions occur under physiologically relevant conditions. Fifth, the current experimental validation was mainly limited to the EdU assay following RRM2 knockdown in PC9 cells. These findings provide preliminary support for the involvement of RRM2 in tumor cell proliferation but do not establish that 4-HNE directly regulates RRM2 or that RRM2 mediates the stromal remodeling features associated with the HNE_high state. Further studies should examine the effects of 4-HNE treatment on RRM2 expression and cellular phenotypes and should incorporate RRM2 knockdown, overexpression, and rescue experiments. Tumor cell–fibroblast coculture models will also be needed to evaluate changes in selected extracellular matrix and stromal remodeling-related factors, including collagen, fibronectin, and laminin.

In summary, this study suggests that 4-HNE-related activity may represent a cellular state linked to oxidative stress, stromal remodeling, and prognostic heterogeneity in lung adenocarcinoma. High HNE activity was associated with fibroblast remodeling, enhanced communication among myeloid, epithelial, and stromal cells, and distinct transcription factor regulatory programs. RRM2 and CYP4B1 represented two different directions within the HNE-related prognostic feature set, with RRM2 showing a clear pro-proliferative role. These findings provide new clues for understanding how lipid peroxidation-related molecular events may contribute to microenvironmental remodeling and prognostic variation in lung adenocarcinoma.

## Data availability statement

The datasets analyzed in this study were obtained from publicly available online databases, and the corresponding sources and accession information are provided in the manuscript. Further information is available from the corresponding author upon reasonable request.

## Ethics statement

This study was based on publicly available datasets and in vitro cell experiments using established cell lines. No human tissue samples, animal experiments, or identifiable personal information were involved. Therefore, ethical approval and informed consent were not required for this study.

## Funding

This study was supported by Joint Funds of the Natural Science Foundation of Tianjin (25JCLMJC00460), Tianjin Key Medical Discipline Construction Project (TJYXZDXK-3-003A).

## CRediT authorship contribution statement

**Pei Zhang:** Conceptualization. **Ke Ma:** Data curation. **Yue Yu:** Formal analysis, Data curation, Conceptualization. **Xu-Chen Cao:** Writing – review & editing, Writing – original draft, Visualization, Validation, Supervision, Software, Resources, Project administration, Methodology, Investigation, Funding acquisition, Formal analysis, Data curation, Conceptualization. **Yang Lyu:** Writing – review & editing, Writing – original draft, Visualization, Validation, Supervision, Software.

## Declaration of competing interest

The authors declare that they have no known competing financial interests or personal relationships that could have appeared to influence the work reported in this paper.

## Appendix Supplementary materials

Image, application 1

Image, application 2
