# Supplementary material for: Integrative single-cell and spatial transcriptomic analysis reveals 4-HNE-related stromal remodeling in lung adenocarcinoma
Source: Transl Oncol. 2026 Aug 29;72:102995. doi: 10.1016/j.tranon.2026.102995 (PMC13545281; doi:10.1016/j.tranon.2026.102995)
Supplement: Supplementary file 1 [file mmc1.docx]

**Supplementary Figure 1. Pseudotime trajectory and dynamic gene expression patterns of fibroblast transcriptional states.** (A) Fibroblast pseudotime trajectories colored by State, fibroblast subtype, pseudotime, HNE group, and tissue source, together with the pseudotime density distribution of HNE_low and HNE_high cells. (B) Heatmap showing the expression patterns of pseudotime-associated genes along the trajectory. (C) Gene expression modules along pseudotime and corresponding GO-BP functional annotations. (D) Expression trends of representative fibroblast-related genes along pseudotime, shown by fibroblast subtype and HNE group.

Supplementary Table S1. Sample-level characteristics of the scRNA-seq dataset used in this study.
